# Clinical characteristics and outcomes of aortic prosthetic valve endocarditis: comparison between transcatheter and surgical bioprostheses

**DOI:** 10.1007/s15010-024-02302-0

**Published:** 2024-06-10

**Authors:** Adrián Jerónimo, Carmen Olmos, Pablo Zulet, Daniel Gómez-Ramírez, Manuel Anguita, Juan Carlos Castillo, Francesc Escrihuela-Vidal, Guillermo Cuervo, Jorge Calderón-Parra, Antonio Ramos, Gonzalo Cabezón, Jesús Álvarez Rodríguez, Paloma Pulido, María de Miguel-Álava, Carmen Sáez, Javier López, Isidre Vilacosta, J. Alberto San Román

**Affiliations:** 1 Instituto Cardiovascular, Hospital Clínico San Carlos, Instituto de Investigación Sanitaria del Hospital Clínico San Carlos (IdISSC), Profesor Martín Lagos, s/n, Madrid, 28040 Spain; 2 Department of Medicine, Faculty of Biomedical and Health Sciences, Universidad Europea de Madrid, Madrid, Spain; 3 Department of Cardiology, Hospital Universitario Reina Sofía. Córdoba, Córdoba, Spain; 4 Instituto Maimónides de Investigación Biomédica, Universidad de Córdoba, Córdoba, Spain; 5 Department of Infectious Diseases, IDIBELL (Institut d´Investigació Biomèdica de Bellvitge), Hospital Universitari de Bellvitge, University of Barcelona, Barcelona, Spain; 6 Department of Internal Medicine, Hospital Universitario Puerta de Hierro, Majadahonda, Madrid, Spain; 7 Department of Cardiology, Hospital Clínico Universitario de Valladolid, Valladolid, Spain; 8 Department of Internal Medicine, Hospital Universitario de La Princesa, Madrid, Spain; 9 Centro de Investigación Biomédica en Red de Enfermedades Cardiovasculares (CIBER-CV), Madrid, Spain; 10 Facultad de Medicina, Universidad Complutense de Madrid, Madrid, Spain

**Keywords:** Infective endocarditis, Bioprosthesis, Transcatheter, TAVI, Cardiac surgery

## Abstract

**Purpose:**

Most data regarding infective endocarditis (IE) after transcatheter aortic valve implantation (TAVI) comes from TAVI registries, rather than IE dedicated cohorts. The objective of our study was to compare the clinical and microbiological profile, imaging features and outcomes of patients with IE after SAVR with a biological prosthetic valve (IE-SAVR) and IE after TAVI (IE-TAVI) from 6 centres with an Endocarditis Team (ET) and broad experience in IE.

**Methods:**

Retrospective analysis of prospectively collected data. From the time of first TAVI implantation in each centre to March 2021, all consecutive patients admitted for IE-SAVR or IE-TAVI were prospectively enrolled. Follow-up was monitored during admission and at 12 months after discharge.

**Results:**

169 patients with IE-SAVR and 41 with IE-TAVI were analysed. Early episodes were more frequent among IE-TAVI. Clinical course during hospitalization was similar in both groups, except for a higher incidence of atrioventricular block in IE-SAVR. The most frequently causative microorganisms were *S. epidermidis*, Enterococcus spp. and *S. aureus* in both groups. Periannular complications were more frequent in IE-SAVR. Cardiac surgery was performed in 53.6% of IE-SAVR and 7.3% of IE-TAVI (p=0.001), despite up to 54.8% of IE-TAVI patients had an indication. No differences were observed about death during hospitalization (32.7% vs 35.0%), and at 1-year follow-up (41.8% vs 37.5%), regardless of whether the patient underwent surgery or not.

**Conclusion:**

Patients with IE-TAVI had a higher incidence of early prosthetic valve IE. Compared to IE-SAVR, IE-TAVI patients underwent cardiac surgery much less frequently, despite having surgical indications. However, in-hospital and 1-year mortality rate was similar between both groups.

**Supplementary Information:**

The online version contains supplementary material available at 10.1007/s15010-024-02302-0.

## Introduction

Prosthetic valve infective endocarditis (IE) is a rare but severe complication after surgical valve replacement, and it is associated with significant morbidity and mortality [1]. Along the last two decades, transcatheter aortic valve implantation (TAVI) has emerged as an effective therapy for aortic stenosis not only in patients deemed at prohibitive risk for surgical aortic valve replacement (SAVR) [2], but also in high [3, 4], intermediate [5, 6] and low surgical-risk patients [7, 8]. Consequently, the number of TAVI procedures has widely increased and is expected to grow exponentially in the coming years, as well as the number of patients at risk for developing IE after TAVI (IE-TAVI).

Based on large TAVI trials and registries, incidence of IE-TAVI and IE after SAVR have been reported to be similar [9–11]. However, these series do not provide detailed information regarding clinical, microbiological, or imaging aspects, so IE-dedicated databases are necessary to achieve further understanding of this disease.

Our study aim was to compare the clinical characteristics, microbiological profile, imaging findings, and outcomes of patients with IE after surgical bioprosthetic aortic valve replacement (IE-SAVR) with those of patients with IE after a TAVI procedure (IE-TAVI).

## Methods

### Patient population

This is a retrospective cohort study assessing prospectively collected data. It has been conducted in 6 IE referral centres in Spain. All centres have dedicated teams for the treatment of IE.

For the purpose of this study, all consecutive patients admitted for IE-SAVR or IE-TAVI from the time of the first TAVI implantation in each centre to March 2021, were prospectively enrolled in a multipurpose registry. To avoid time-dependent bias, those patients with a diagnosis of IE-SAVR prior to the implementation of TAVI in their belonging centre were systematically excluded.

This study was performed in line with the principles of the Declaration of Helsinki. The protocol was approved by the local ethics committee at every site and patients’ informed consent was waived because it involved only the analysis of data obtained during standard clinical practice.

All patients were evaluated and treated by the local Endocarditis Teams (ET) during hospitalization, undergoing a thorough diagnostic work-up which included a detailed clinical history and physical examination, electrocardiography, blood analysis, blood cultures at admission and 48–72 h after the initiation of antibiotic therapy, and transthoracic (TTE) and/or transesophageal echocardiography (TEE). Other imaging tests, such as positron emission tomography/computed tomography (PET/CT), abdominal ultrasound, computed tomography (CT), magnetic resonance or cerebral arteriography were performed at ET’s discretion. An initial classification of episodes as rejected, possible, and definite IE was established, according to the modified Duke criteria [12] until 2015 and the ESC modified diagnostic criteria [13] thereafter. Episodes were considered early prosthetic valve IE when diagnosed within the first 12 months after valve implantation [13]. Likewise, indications for cardiac surgery were stablished by the ET according to standing ESC guidelines. All patients were followed-up at 12 months after hospital discharge.

Information regarding prosthetic valve implantation and follow-up until the admission for the IE episode was collected from hospital records.

### Statistical analysis

Quantitative variables were expressed as median and interquartile range (IQR) or mean and standard deviation (SD). Categorical variables were expressed as frequencies and percentages. To compare qualitative variables, the χ2 test or Fisher’s exact test were used. Quantitative variables were compared using Student’s t-test and its non-parametric equivalent Mann-Whitney U test. Assessment of normality and equality of variances for continuous data was performed using the Shapiro–Wilk test and the Levene test, respectively.

Logistic regression analysis was performed to identify independent predictors of death during hospitalization in both groups. Variables statistically significant in the univariable analysis or considered clinically relevant were included in a multivariable regression model. The adjusted odds ratios (ORs) with 95% confidence intervals (Cis) for each variable were calculated.

All tests were two-sided and differences were considered statistically significant at p-values < 0.05. Statistical analyses were performed with Stata/IC12.1 (StataCorp, College Station, Texas, USA).

## Results

### Baseline characteristics of the study population at IE diagnosis

A total of 169 patients with EI-SAVR and 41 with EI-TAVI were analysed. Patients with EI-TAVI were significantly older (80.3 (76.7–83.4) vs. 76.6 (69.9–80.2) years old, *p* = 0.001) and presented a higher prevalence of diabetes, chronic obstructive pulmonary disease, and chronic anaemia, as well as higher scores in the Frail scale and Charlson index. No differences were observed in the proportion of patients with a previous episode of IE. Other population characteristics at the time of admission for IE are summarized in Table 1.

**Table 1:** Baseline characteristics at the time of admission for IE

	IE-SAVR (*n* = 169)	IE-TAVI (*n* = 41)	*p*
Age (years)	76.6 (69.9–80.2)	80.3 (76.7–83.4)	**0.001**
Female sex	55 (32.5)	14 (34.2)	0.845
Diabetes	41 (24.3)	22 (53.7)	**0.001**
BMI (kg/m^2^)	27.7 (24.6–30.5)	25.6 (23.7–28.4)	**0.043**
Chronic anaemia	46 (27.2)	19 (46.3)	**0.018**
Chronic kidney disease	43 (25.4)	13 (31.7)	0.416
Immunodepression	23 (13.6)	6 (14.6)	0.865
Active neoplasia	18 (10.7)	4 (9.8)	0.867
COPD / asthma	24 (14.2)	13 (35.1)	**0.008**
Collagenopathies	5 (3.0)	0 (0)	0.265
Other prosthetic valves	15 (8.9)	1 (2.4)	0.163
Cardiac implantable electronic devices carriers	29 (17.2)	4 (9.8)	**0.024**
Previous AMI	20 (11.8)	6 (14.6)	0.621
Previous revascularization	34 (23.1)	7 (18.9)	0.275
Previous IE			
• Reinfection	19 (11.3)	2 (4.9)	0.162
• Relapse	2 (1.2)	0 (0)	
Atrial fibrillation	58 (34.3)	18 (43.9)	0.252
Chronic anticoagulation	61 (39.1)	19 (50.0)	0.221
Anticoagulation during hospitalization	67 (43.0)	19 (50.0)	0.433
Frail scale	0 (0–2)	2.5 (1–3)	**0.001**
Charlson index	5 (3–7)	6 (5–8)	**0.001**
Barthel index	100 (90–100)	100 (80–100)	0.317

Values are presented as frequency and percentage or median and interquartile ranges. Bold values are significant

AMI: acute myocardial infarction; BMI: body mass index; COPD: chronic obstructive pulmonary disease; IE: infective endocarditis; IE-SAVR: IE after surgical aortic valve replacement with a biological prosthetic valve; IE-TAVI: IE after transcatheter aortic valve implantation

Data regarding index prosthetic valve implantation and periprocedural complications are presented in Online Resource 1–5. Echocardiographic follow-up after valve implantation and before admission for IE showed a significantly higher proportion of periprosthetic valve regurgitation in those patients undergoing TAVI. However, in most of the cases it was graded as mild (Online Resource 6).

### Epidemiological characteristics

Early prosthetic valve IE was significantly more frequent in IE-TAVI than in IE-SAVR (78.1% vs. 39.3%; *p* = 0.001). Likewise, time elapsed from the procedure (percutaneous/surgical) until admission for IE was shorter among IE-TAVI (0.6 (0.2-1.0) vs. 1.6 (0.6–5.1) years, *p* = 0.001).

In both groups, 90% of the patients were diagnosed with definite IE; the rest had possible IE. A significantly larger proportion of episodes from IE-SAVR were referred from another hospital. Regarding potential portals of entry, no differences were observed among groups, except for a higher prevalence of endovascular catheters (peripheral or central venous lines and arterial catheters) within the previous 3 months to IE diagnosis in IE-TAVI (22.0% vs. 9.5%, *p* = 0.027). Time from admission to IE diagnosis was longer among patients with IE-TAVI (1.5 (0–6) vs. 0 (0–3) days, *p* = 0.018). Other details regarding IE diagnosis are summarized in Table 2.

**Table 2:** Diagnosis of IE

	IE-SAVR (*n* = 169)	IE-TAVI (*n* = 41)	*p*
Time from prosthetic valve implantation (years) • Early IE	1.6 (0.6-5-1) 66 (39.3)	0.6 (0.2-1.0) 32 (78.1)	**0.001** **0.001**
Major criteria			
• Positive BC	152/168 (90.5)	39/41 (95.1)	0.342
• Positive echocardiography	137/167 (82.0)	30/41 (73.2)	0.201
• Positive PET/CT or SPECT/CT	32/59 (54.2)	12/17 (70.6)	0.098
• Positive CT	4/167 (2.4)	3/41 (7.3)	**0.001**
Minor criteria			
• Predisposition (heart condition)	168 (100.0)	41 (100.0)	0.999
• Fever (temperature > 38ºC) • Vascular phenomena	147 (88.6) 59 (35.1)	37 (90.2) 17 (41.5)	0.758 0.449
• Vascular phenomena	59 (35.1)	17 (41.5)	0.449
• Immunological phenomena	4 (2.4)	1 (2.4)	0.983
• Positive BC	2 (1.2)	0 (0)	0.481
Definite vs. possible IE			
• Definite	154 (91.1)	36 (87.8)	0.516
• Possible	15 (8.9)	5 (12.2)	
Referred from another hospital	80 (47.4)	9 (22.0)	**0.003**
Origin			
• Community acquired	91 (53.9)	18 (43.9)	0.092
• Healthcare-associated *			
- Non-nosocomial	40 (23.7)	7 (17.1)	
- Nosohusial	38 (22.5)	16 (39.0)	
Potential portals of entry			
• Gastrointestinal tract intervention ^a, b^	11 (6.5)	5 (12.2)	0.218
• Peripheral or central venous lines and arterial catheters ^a^	16 (9.5)	9 (22.0)	**0.027**
• Dental procedure ^a^	5 (3.0)	2 (4.9)	0.437
• Local infection	14 (8.3)	2 (4.9)	0.388
• Cardiac surgery ^a^	2 (1.2)	0 (0)	0.575
• Non-cardiac surgery ^a^	4 (2.4)	0 (0)	0.344
• Other	5 (3.0)	1 (2.4)	0.421

Values are presented as frequency and percentage or median and interquartile ranges. Bold values are significant

BC: blood cultures; CT: computed tomography; IE: infective endocarditis; IE-SAVR: IE after surgical aortic valve replacement with a biological prosthetic valve; IE-TAVI: IE after transcatheter aortic valve implantation; PET: positron emission tomography; SPECT: single photon emission computerized tomography

*Healthcare-associated IE was defined as IE manifesting > 48 h after hospital admission or IE associated with significant invasive procedures performed 6 months prior to clinical diagnosis. It includes nosocomial (hospital-acquired) as well as nosohusial infections (acquired in other healthcare settings, such as hemodialysis, nursing homes, or day hospitals)

^a^Within the three months prior to the diagnosis of IE

^b^Surgery or endoscopic procedures (with or without biopsy sampling)

### Clinical presentation

**Fig. 1:**
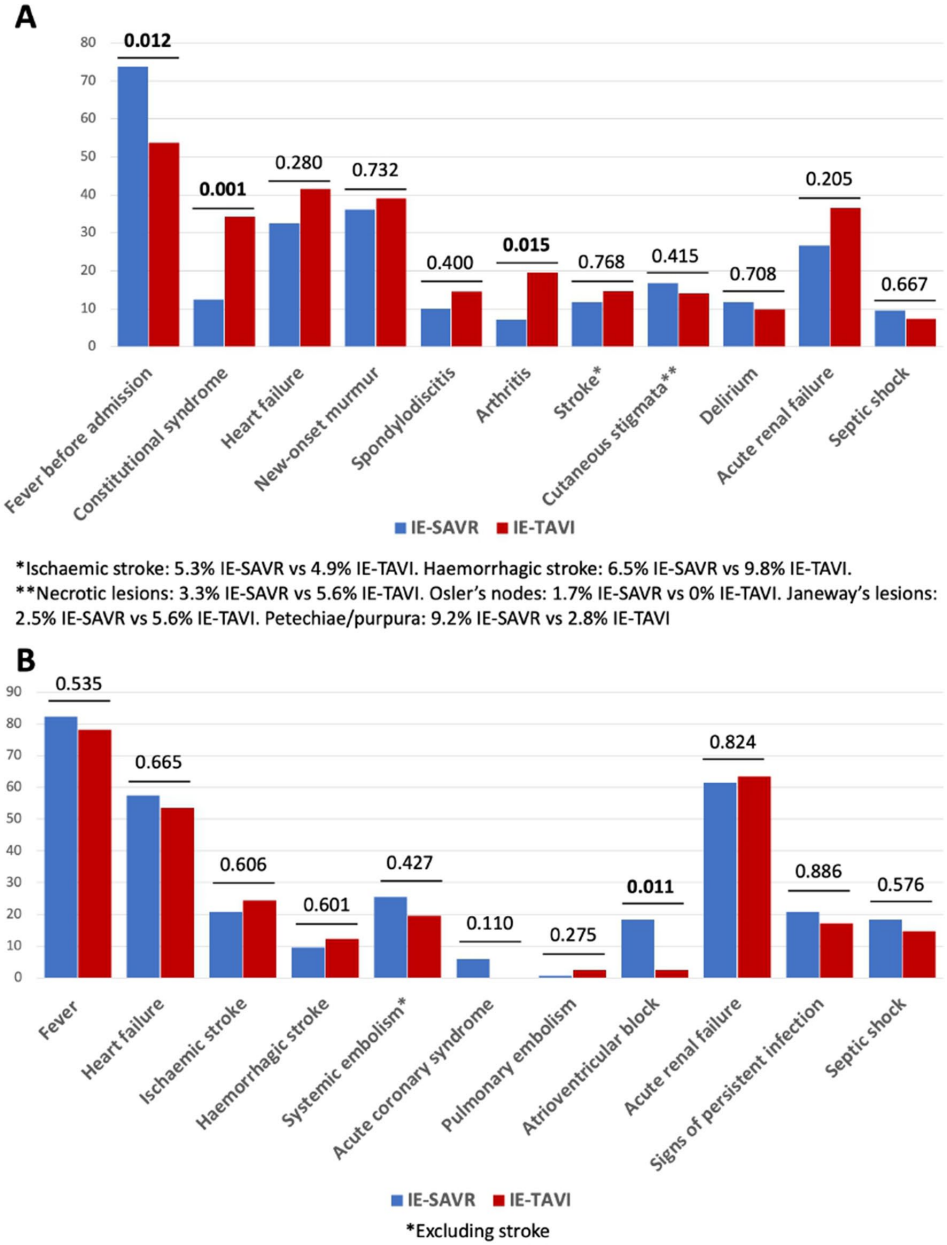
Clinical information at admission (**A**) and during hospitalization for IE (**B**). Values are presented as percentage. Bold values are significant. IE: infective endocarditis; IE-SAVR: IE after surgical aortic valve replacement with a biological prosthetic valve; IE-TAVI: IE after transcatheter aortic valve implantation

Patients with IE-TAVI less frequently had fever before admission and more frequently presented with constitutional syndrome and reactive arthritis. No significant differences in other signs or symptoms were observed either at admission or during hospitalization, except for atrioventricular block, which was more incidence among IE-SAVR (Fig. 1). Time from hospital admission to IE diagnosis was longer in IE-TAVI (1.5 vs. 0 days; *p* = 0.018), although no differences in time from the onset of symptoms or length of hospital stay were observed.

### Microbiology

The proportion of positive blood cultures at admission (95.8% in IE-SAVR vs. 97.6% in IE-TAVI; *p* = 0.605) and 48–72 h after the initiation of antibiotic treatment (28.0% vs. 24.4%; *p* = 0.367) were comparable in both groups.

The most frequently isolated microorganisms in both groups were *Staphylococcus epidermidis* (26.6% in IE-SAVR, 26.8% in IE-TAVI), *Enterococcus* spp. (18.3% vs. 24.4%) and *Staphylococcus aureus* (11.2% vs. 22.0%). No significant differences in the microbiological profile were observed between both groups, except for a trend towards higher proportion of *S. aureus* infection in patients with IE-TAVI (*p* = 0.070) (Online Resource 7).

### Imaging tests findings

By echocardiography, no differences were observed regarding the detection of vegetations and their size. Nevertheless, periannular complications were more frequently identified in IE-SAVR (44.4% vs. 19.5%; *p* = 0.003), and abscess size was larger (15 mm (9–22) vs. 6 mm (5–8); *p* = 0.002). Similar proportions of intra- and peri-prosthetic valve regurgitation were observed in both groups (Table 3). Vegetations were numerically more frequent in balloon-expandable TAVI compared to self-expandable ones (73.3% vs. 54.6%), although no statistically significant differences were observed, neither regarding vegetation size or periannular complications (Online Resource 8).

**Table 3:** Echocardiographic findings

	IE-SAVR (*n* = 169)	IE-TAVI (*n* = 41)	*p*
Time from the beginning of symptoms to 1st TTE (days)	1 (0–7)	4 (1–8)	0.145
Number of TTE during hospitalization	1 (1–2)	1 (1–2)	**0.007**
Time from the beginning of symptoms to 1st TOE (days)	3 (1–8)	4 (1–10)	0.138
Number of TOE during hospitalization	2 (1–3)	1 (1–2)	0.593
Vegetations	118 (70.2)	26 (63.4)	0.397
Vegetation echogenicity			
• Thrombus-like	25 (41.7)	10 (47.6)	0.655
• Similar to myocardium	33 (55.0)	11 (52.4)	
• Calcium-like	2 (3.3)	0 (0)	
Vegetation morphology			
• Sessile	15 (23.8)	7 (29.2)	0.736
• Pedunculated	47 (74.6)	17 (70.8)	0.736
Vegetation diameter (mm)	11 (7–15)	12 (8–17)	0.375
Periannular complications	75 (44.4)	8 (19.5)	**0.003**
• Abscess	64 (38.1)	7 (17.1)	**0.011**
• Abscess diameter (mm)	15 (9–22)	6 (5–8)	**0.002**
• Pseudoaneurysm	18 (10.8)	2 (4.9)	0.251
• Pseudoaneurysm diameter (mm)	15 (10–27)	6.8 (3.5–10)	0.112
Leaflet perforation	12 (7.1)	1 (2.4)	0.264
Valvulitis	25 (14.9)	5 (12.2)	0.660
Moderate or severe prosthetic valve stenosis	46 (27.4)	5 (12.2)	**0.042**
Intra-prosthetic valve regurgitation			
• Mild	30 (18.0)	5 (12.2)	0.810
• Moderate	17 (10.2)	5 (12.2)	
• Severe	10 (6.0)	2 (4.9)	
Peri-prosthetic valve regurgitation			
• Mild	8 (4.8)	5 (12.2)	0.248
• Moderate	17 (10.2)	6 (14.6)	
• Severe	17 (10.2)	3 (7.3)	
LVEF (%)	60 (54–65)	60 (50–64)	0.475
Pulmonary hypertension	68 (40.7)	20 (50.0)	0.299
Pericardial effusion	50 (30.1)	6 (15.0)	0.054

Values are presented as frequency and percentage or median and interquartile ranges. Bold values are significant

IE: infective endocarditis; IE-SAVR: IE after surgical aortic valve replacement with a biological prosthetic valve; IE-TAVI: IE after transcatheter aortic valve implantation; LVEF: left ventricle ejection fraction; TOE: transoesophageal echocardiography; TTE: transthoracic echocardiography

IE-related findings on other imaging techniques are summarized on Online Resource 9, as well as the proportion of other structures compromised by IE apart from the valve itself (Online Resource 10).

### Treatment

No differences were observed regarding antibiotic treatment duration. Cardiac surgery was more frequently performed in IE-SAVR (53.6% vs. 7.3%; *p* = 0.001). Besides, the proportion of patients with an indication for surgery was higher in this group (78.7% vs. 53.7%; *p* = 0.006). Nevertheless, the percentage of patients who did not undergo cardiac surgery despite presenting one or more indications, due to high operative risk, was higher among IE-TAVI (26.3% vs. 47.5%; *p* = 0.009).

Indications for surgery as well as reasons for not performing surgery despite being indicated are presented in Table 4.

**Table 4:** Treatment of IE episodes

	IE-SAVR (*n* = 169)	IE-TAVI (*n* = 41)	*p*
Duration of antibiotic treatment (days)	42 (29–53)	43 (32–51)	0.642
Outpatient antibiotic treatment administration	41 (24.3)	13 (31.7)	0.134
Outpatient antibiotic treatment duration (days)	27 (15–34)	29 (15–48)	0.718
EuroScore II	12.9 (7.4–33)	11.7 (5.0-44.7)	0.850
Risk-E Score	35.1 (24.2–49.7)	39.6 (31-54.2)	0.132
Cardiac surgery performed	91^a^ (53.6)	3^b^ (7.3)	**0.001**
Surgical indications in patients undergoing surgery			
• Heart failure	19 (21.1)	0 (0)	**0.014**
• Prosthetic dysfunction • Single embolism	15 (16.7) 1 (1.1)	1 (33.3) 0 (0)	
• Recurrent embolism	2 (2.2)	1 (33.3)	
• Large vegetation	3 (3.3)	1 (33.3)	
• Signs of persistent infection	10 (11.1)	0 (0)	
• Periannular complications	40 (44.4)	0 (0)	
Surgery timing			
• Emergent	22 (20.4)	0 (0)	0.658
• Urgent	70 (64.8)	3 (100.0)	
• Elective	16 (14.8)	0 (0)	
Time from IE diagnosis to surgery (days)	8.5 (4–19)	7 (1–33)	0.802
Type of surgery			
• Biological prosthetic valve	57 (63.3)	2 (66.7)	0.996
• Mechanical prosthetic valve	28 (31.1)	1 (33.3)	
• Homograft	1 (1.1)	0 (0)	
• Composite graft	3 (3.3)	0 (0)	
Indication for surgery	133 (78.7)	22 (53.7)	**0.006**
Indication for surgery, but not performed	42^c^ (26.3)	19^d^ (47.5)	**0.009**

Values are presented as frequency and percentage or median and interquartile ranges. Bold values are significant

IE: infective endocarditis; IE-SAVR: IE after surgical aortic valve replacement with a biological prosthetic valve; IE-TAVI: IE after transcatheter aortic valve implantation

^a^Including 28 mechanical prosthetic valves, 57 biological prosthetic valves, 1 homograft and 3 tube grafts

^b^1 mechanical and 2 biological prosthetic valves

^c^38 cases for high surgical risk, 1 case due to patient’s decision, 3 cases for death before surgery

^d^High surgical risk in all of the cases

### Outcomes

All-cause and IE-related death rates during hospitalization and at 12-month follow-up were comparable in both groups, regardless of patients undergoing cardiac surgery or not. No differences were observed either in the incidence of IE relapse, or valve surgery, or admission for heart failure during follow-up (Table 5).

**Table 5:** Outcomes of IE episodes

	IE-SAVR (*n* = 169)	IE-TAVI (*n* = 41)	*p*
All-cause death during hospitalization	55 (32.7)	14 (35.0)	0.785
Causes of death during hospitalization			
- Septic shock	12 (19.7)	6 (42.9)	0.067
- Heart failure	15 (24.6)	5 (35.7)	0.432
- Inability to withdraw extracorporeal circulation	7 (11.5)	0 (0)	0.248
- Stroke	5 (8.2)	1 (7.1)	0.356
- Haemorrhagic shock	2 (3.3)	0 (0)	0.454
- Multiorgan failure	9 (14.8)	2 (14.3)	0.687
- Other	5^a^ (8.2)	0 (0)	0.312
All-cause death at 1-year follow-up	69 (41.8)	15 (37.5)	0.618
IE-related death at 1-year follow-up	57 (89.1)	15 (100.0)	0.180
Relapse at 1-year follow-up	8 (5.9)	1 (3.7)	0.645
Valvular surgery at 1-year follow-up	17 (12.6)	2 (7.4)	0.445
Admission for heart failure at 1-year follow-up	15 (11.1)	5 (19.2)	0.250
**Patients undergoing surgery**:	**91 (53.6)**	**3 (7.3)**	
- All-cause death during hospitalization	24 (26.4)	1 (33.3)	0.788
- All-cause death at 1-year follow-up	31 (34.1)	1 (33.3)	0.979
**Patients not undergoing surgery**:	**78 (46.2)**	**38 (92.7)**	
- All-cause death during hospitalization	31 (40.3)	13 (35.1)	0.599
- All-cause death at 1-year follow-up	38 (51.4)	14 (37.8)	0.179

Values are presented as frequency and percentage or median and interquartile ranges. Bold values are significant

IE: infective endocarditis; IE-SAVR: IE after surgical aortic valve replacement with a biological prosthetic valve; IE-TAVI: IE after transcatheter aortic valve implantation

^a^1 case of massive lung collapse; 1 case of SARS-CoV-2 pneumonia; 1 case of metastatic encephalitis; 1 case of hematemesis, respiratory failure and complete atrioventricular block; 1 case of aspiration pneumonia

A subanalysis focused on patients with surgical indications who did not undergo surgery showed high all-cause death rates during hospitalization, similar among IE-SAVR and IE-TAVI (68.3% vs. 63.2%; *p* = 0.695). Interestingly, only one patient with IE-TAVI who survived the index hospitalization died during follow-up. Thus, considering only patients discharged alive, 1-year mortality was low in both groups (12.7 vs. 4.0%; *p* = 0.210).

Additional information regarding the subset of patients with IE-TAVI with a surgical indication who did not undergo surgery is summarized in Online Resource 11. As expected, in this group of patients, heart failure and septic shock were significantly more frequent in patients who died during the index hospitalization.

To further evaluate outcomes predictors, a logistic regression analysis for in-hospital mortality in both groups was performed. In IE-SAVR, variables independently associated with all-cause mortality during hospitalization were older age, heart failure and septic shock, whereas undergoing surgical treatment was a protective factor. On the other hand, periannular complications and heart failure were the only independent predictors of death during hospitalization in IE-TAVI (Table 6).

**Table 6:** Multivariable analysis to identify predictors of death during hospitalization in both groups

Variables	OR (95% CI)	*p*
**IE-SAVR** ^a^		
Age	1.1 (1.0-1.2)	0.001
Heart failure	6.04 (2.2–16.6)	0.001
Septic shock	21.8 (6.4–73.4)	0.001
Surgery	0.4 (0.1–0.9)	0.026
**IE-TAVI** ^**b**^		
Periannular complications	14.2 (1.2–174.0)	0.037
Heart failure	15.2 (1.2-199.1)	0.038

CI: confidence interval; IE: infective endocarditis; IE-SAVR: IE after surgical aortic valve replacement with a biological prosthetic valve; IE-TAVI: IE after transcatheter aortic valve implantation; OR: odds ratio

^a^Variables included in the multivariable logistic regression were: age, COPD/asthma, previous revascularization, Charlson index, atrioventricular block, acute kidney disease, septic shock, heart failure, *S. aureus* as causing microorganism and surgery

^b^Variables included in the multivariable logistic regression were: periannular complications, heart failure, septic shock and definite/possible IE

## Discussion

The present study comparatively displays the clinical, microbiological, and imaging characteristics of IE-SAVR and IE-TAVI, as well as their outcomes in 210 consecutive cases from tertiary referral centres with dedicated ET. The most relevant findings include: (1) patients with IE-TAVI were significantly older, more fragile, and with more comorbidities, compared to IE-SAVR; (2) early IE was more frequent amongst IE-TAVI; (3) the microbiological profile of both entities was comparable, except for a higher proportion of *S. aureus* infection in IE-TAVI; (4) abscesses were more frequent and larger in IE-SAVR, and these patients developed more frequently atrioventricular block; (5) regarding clinical presentation, IE-TAVI patients had more frequently constitutional symptoms; (6) the proportion of patients who did not undergo surgery in spite of having a surgical indication was higher in IE-TAVI; and (7) mortality during hospitalization and follow-up was similar in both groups.

Despite TAVI procedure has become less invasive and its indication has expanded to lower-risk patients, the incidence of IE-TAVI has remained stable along the years [14]. Initially, it was described that the incidence of IE was higher in TAVI, but recent large observational studies have shown no significant differences in comparison with SAVR [9, 11, 15]. On the other hand, and as it is shown in our cohort, the risk of early prosthetic valve IE is known to be higher in TAVI [15], with the highest incidence occurring during the first seven months after valve implantation [16]. In the Swiss TAVI registry, early IE episodes were significantly more common than late ones, and the highest incidence was noted in the first 100 days following valve implantation. These findings correspond to a risk of IE six times higher in the first 100 days after the procedure than after the first year [17]. Nevertheless, in a recent study which compared a historical cohort of TAVI patients with a contemporary one in which minimalist approaches are aimed, Del Val et al. observed a decline in early IE episodes in recent years, particularly in those occurring within the first 2 months after the procedure [18].

In high-income countries, IE is a disease which predominantly affects male older adults [19]. In our cohort, as expected, a predominant proportion of male sex was observed in both groups. We also found that patients with IE-TAVI had more comorbidities, such as diabetes, pulmonary diseases, and chronic anemia. Likewise, significant residual periprosthetic regurgitation and vascular complications, which are already known procedure-related risk factors for IE-TAVI, were more frequent in our IE-TAVI patients, compared to IE-SAVR [20].

In relation to all the above, and as previously described in other series [18, 20], a very high prevalence of healthcare associated cases was observed among IE-TAVI, although non-significant differences were found when compared to IE-SAVR, due to the limited sample size. We observed a higher proportion of cases referred from another hospital among IE-SAVR, which may be explained by the fact that these patients are more often considered surgical candidates, due to a lower operative risk.

Enterococci have been reported as a very common cause of IE-TAVI, due to their affinity for the groin region and the widespread use of transfemoral access for TAVI implantation [21]. In IE-SAVR, enterococci are less frequently isolated, although an increasing trend of their incidence as a cause of IE has been described within the last two decades [19]. In our cohort, coagulase-negative staphylococci, enterococci, and *Staphylococcus aureus* were the most frequently isolated microorganisms in both groups, which is consistent with prior studies [11]. We did not find significant differences in the proportion of enterococcal infections, but *S. aureus* was twice as frequent in IE-TAVI patients, compared to those with IE-SAVR.

Regarding clinical features, fever was the most frequent sign in both groups. Nonetheless, and in accordance with previous series, we found that TAVI patients, despite having a higher proportion of *S. aureus* infections, presented less frequently with fever, and had proportionally higher prevalence of nonspecific symptoms, such as constitutional syndrome and arthritis [18, 20]. These findings may be related to the fact that IE-TAVI occurs in older patients with a higher comorbidity burden and may be one of the reasons that led to the observed delay in diagnosis [14].

No significant differences were observed regarding heart failure or systemic embolisms at admission or during hospitalization between both groups of patients. Conversely, IE-SAVR patients developed more frequently atrioventricular blocks, and presented more and larger periannular complications, as recently reported by Panagides et al [22]. Contrary to our findings, some studies have described that the incidence of periannular complications is similar in both IE-SAVR and IE-TAVI patients, or even more frequent among the latter [14, 23, 24]. In a recent systematic review including 107 IE-TAVI cases who underwent surgery, Malvindi et al. found that 34% of episodes had annular abscesses [25]. Nevertheless, these results may be influenced by selection bias, as patients with periannular complications are more likely to be treated surgically.

Regarding patient’s management, the benefit of surgery in both native and prosthetic valve IE in patients with surgical indications is firmly endorsed by a large body of evidence [26]. However, the optimal therapeutic approach for IE-TAVI is not well established yet. Due to advanced age and comorbidities of patients undergoing TAVI, the proportion of patients receiving surgery in the context of IE, despite having clear indications, is consistently low (< 20%) [9, 18, 20]. Moreover, some studies assessing the outcomes of cardiac surgery for the treatment of IE-TAVI have shown no benefit in terms of survival during admission and at one-year follow-up [20, 27–29]. In our cohort, only 3 (7.3%) patients with IE-TAVI underwent cardiac surgery, and the survival rate of IE-TAVI patients who underwent surgery and those who were treated conservatively was similar.

On the other hand, a meta-analysis by Tinica et al. found that surgical treatment was associated with a lower mortality rate (OR 0.15, 95% CI 0.04 to 0.62), although they noticed the possibility of publishing bias [16]. In addition, Panagides et al. did observe a higher survival proportion with surgical treatment among patients with IE-TAVI and periannular complications [30]. Moreover, Saha et al. reported a survival to discharge rate of 88.4% in low-intermediate risk IE-TAVI patients who underwent surgery [31]. Therefore, surgical risk and patient selection are crucial when evaluating the results of surgery in this singular group of patients.

When comparing the outcomes between IE-TAVI and IE-SAVR, large national-based registries have reported a significantly higher in-hospital mortality in IE-TAVI patients compared to IE-SAVR, ascribing it to a more frequently conservative treatment strategy applied in a population with a higher age and more comorbidities. Of note, both, the proportion of patients with surgical indications and the percentage of those who actually undergo surgery, are often missing from these datasets [11, 15]. On the other hand, Moriyama et al. described no differences regarding in-hospital death between both groups of patients (20.0% in IE-TAVI vs. 32.1% in IE-SAVR, *p* = 0.44), although a lower proportion of TAVI patients received surgery [32]. In addition, Panagides et al. reported similar 1-year mortality rates among IE-TAVI and IE-SAVR [22]. Likewise, in our cohort, no differences in in-hospital and 1-year mortality rates were observed, despite the unbalanced proportion of surgery among groups.

We have not found definite reasons to explain this relatively “more benign” course in the case of IE-TAVI. Yet, we must bear in mind that patients with IE-TAVI had less frequently an indication for surgery, compared to the IE-SAVR group, and presented fewer periannular complications. In addition, 50% of patients with IE-SAVR in our series who underwent surgery presented periannular complications or signs of persistent infection, uncontrolled infection being the IE surgical indication associated to the highest mortality [33]. Therefore, it should not be surprising that these patients had poor outcomes, despite undergoing surgery.

In the setting of IE in elderly patients, Lopez-Wolf et al. observed a lower proportion of fever, less severe heart failure, a lower incidence of vegetations and abscesses, fewer surgical indications and a lower mortality among octogenarians with IE, in comparison with younger patients [34]. Furthermore, data from the ESC EORP EURO-ENDO registry showed that octogenarian patients with EI presented surgical indications less frequently than younger adults. Besides, among octogenarians, mortality was lower in patients without any indications for surgery, compared to those with surgical indications [35]. Conversely, other groups have reported better outcomes in elderly patients who were surgically treated [36].

Therefore, in the absence of clear evidence, patient selection, dedicated care and close follow-up by experienced ET become of capital importance when treating elderly patients. On this regard, the use of prolonged antibiotic therapy in our patients who did not undergo surgery may have played a role in the relatively low mortality and satisfactory evolution after discharge of the IE-TAVI group [37].

Our study has several limitations inherent to its observational design. Firstly, all participating centres were high-volume hospitals with experienced ET, so referral and selection bias may be present. Patients with severe comorbidities and considered not surgical candidates are less likely to be transferred to referral hospitals, and this could have occurred more frequently in patients with IE on TAVI. Secondly, since not all patients undergoing SAVR and TAVI in the participating centres were included, incidence of IE in both groups cannot be calculated. Thirdly, theoretical surgical indications of patients who did not undergo surgery are not available. Finally, the limited sample size, especially due to the low number of IE-TAVI cases, does not allow to formulate definite conclusions. Nevertheless, and contrary to large series comparing SAVR and IE-TAVI, all our data come from prospective cohorts of patients, from referral centres for IE with dedicated ET, which provide reliable information regarding clinical, microbiological, and imaging features.

## Conclusions

Patients with IE-TAVI were older, with more comorbidities, and had a higher incidence of early IE. Compared to IE-SAVR, IE-TAVI patients underwent cardiac surgery much less frequently, despite having surgical indications. However, in-hospital and 1-year mortality were similar in both groups.

## Electronic supplementary material

Below is the link to the electronic supplementary material.

Supplementary Material 1

## Data Availability

Data is provided within the manuscript and supplementary information files.
