# Supplementary material for: Clinical characteristics and outcomes of aortic prosthetic valve endocarditis: comparison between transcatheter and surgical bioprostheses
Source: Infection. 2024 Jun 10;52(6):2425–34. doi: 10.1007/s15010-024-02302-0 (PMC11621159; doi:10.1007/s15010-024-02302-0)
Supplement: Supplementary file 1 — Supplementary Material 1 [file 15010_2024_2302_MOESM1_ESM.docx]

**SUPPLEMENTARY INFORMATION**

**Online Resource 1. Baseline characteristics of both groups at the moment of valve implantation.**

|  | **SAVR**  **(n=169)** | **TAVI**  **(n=41)** | **p** |
| --- | --- | --- | --- |
| Age at the time of the procedure | 72.9 (67.1-77.5) | 76.6 (69.9-80.2) | **0.001** |
| Indication   - Severe AS - Severe AR - Double aortic lesion♦ - PVD - Native valve IE - Prosthetic valve IE | 103 (64.0)  28 (17.4)  13 (8.1)  3 (1.9)  10 (6.2)  4 (1.9) | 38 (92.7)  1 (2.4)  0 (0)  2 (4.9)  0 (0)  0 (0) | **0.004** |
| Prosthetic valve diameter (mm) | 23 (21-23) | 26 (25-29) | **0.001** |
| Timing   - Emergent - Urgent - Elective | 3 (1.9)  22 (14.0)  132 (84.1) | 1 (2.4)  8 (19.5)  32 (78.1) | 0.658 |
| Hospitalization duration (days) | 10 (7-18) | 9 (5-16) | 0.130 |
| Within 48 hours before the procedure | | | |
| Heart failure | 26 (17.7) | 12 (30.0) | 0.086 |
| Respiratory infection / Pneumonia | 2 (1.4) | 1 (2.5) | 0.611 |
| Urinary tract infection | 1 (0.7) | 0 (0) | 0.601 |
| Skin / Soft tissue infection | 0 (0) | 0 (0) | - |
| Other infections | 9 (6.1) | 1 (2.5) | 0.367 |
| Antibiotic treatment | 13 (8.8) | 2 (4.9) | 0.428 |
| During the procedure / After the procedure and before hospital discharge | | | |
| Endotracheal intubation | 155 (100.0) | 11 (27.5) | **0.001** |
| Blood product transfusion | 66 (51.2) | 9 (22.5) | **0.005** |
| Temporary cardiac pacing after the procedure | 72 (49.0) | 16 (40.0) | 0.313 |
| Permanent pacemaker implantation | 10 (6.8) | 4 (9.8) | 0.214 |
| Shock / vasoactive drugs requirement | 41 (28.5) | 2 (5.1) | **0.008** |
| Acute heart failure | 29 (20.1) | 7 (18.0) | 0.760 |
| Fever | 18 (12.4) | 6 (15.4) | 0.625 |
| Vascular access complications   - Fistula / Pseudoaneurysm - Haematoma - Infection | 0 (0)  4 (2.8)  1 (0.7) | 2 (5.1)  6 (15.4)  0 (0) | **0.006**  **0.002**  0.603 |
| Surgical wound infection and mediastinitis | 6 (4.1) | - | - |
| Respiratory infection / Pneumonia | 16 (11.0) | 3 (7.7) | 0.543 |
| Urinary tract infection | 8 (5.5) | 2 (5.1) | 0.924 |
| Skin / Soft tissue infection | 0 (0) | 0 (0) | - |
| Other infections | 8 (5.6) | 5 (12.8) | 0.117 |
| Antibiotic treatment | 34 (23.3) | 9 (23.1) | 0.978 |

Values are presented as frequency and percentage or median and interquartile ranges. Bold values are significant.

AR: aortic regurgitation; AS: aortic stenosis; IE: infective endocarditis; PVD: prosthetic valve dysfunction; SAVR: surgical aortic valve replacement; TAVI: transcatheter aortic valve implantation.

**Online Resource 2. Prosthetic valve types used in the index procedure**

SAVR

| **Stented bioprosthetic valves** | |
| --- | --- |
| Sorin Mitroflow | 49 |
| Carpentier-Edwards Perimount | 40 |
| St Jude Medical Trifecta | 21 |
| Sorin Crown PRT | 4 |
| Carpentier-Edwards Magna Ease | 3 |
| Labcor TBLP Supra valve | 2 |
| Medtronic Hancock II | 2 |
| Edwards Intuity Elite | 1 |
| Carpentier-Edwards Magna | 1 |
| Medtronic Avalus | 1 |
| St Jude Medical Biocor | 1 |
| *Sutureless* | |
| Sorin Perceval | 4 |
| **Stentless bioprosthetic valves** | |
| Sorin Freedom Solo | 2 |
| Edwards Prima Plus | 1 |
| *Sutureless* | |
| Medtronic 3f Enable | 4 |

TAVI

| **Balloon-expandable** | 15 (37.5) |
| --- | --- |
| Edwards Sapien  Edwards XT  Edwards S3 | 4 (10.0)  1 (2.5)  10 (25.0) |
| **Self-expanding** | 22 (55.0) |
| Medtronic CoreValve | 10 (25.0) |
| Medtronic Evolut-R | 1 (2.5) |
| Medtronic Evolut-Pro | 4 (10.0) |
| St Jude Portico | 4 (10.0) |
| Symetis Acurate | 3 (7.5) |
| **Other designs** | |
| Boston Lotus Edge | 3 (7.5) |

SAVR: surgical aortic valve replacement; TAVI: transcatheter aortic valve implantation.

**Online Resource 3. Prosthetic valve diameters used in the index procedure.**

SAVR

| 19 mm  20 mm  21 mm  22 mm  23 mm  24 mm  25 mm  26 mm  27 mm  28 mm  29 mm | 11 (7.6)  1 (0.7)  49 (34.0)  0 (0)  53 (36.8)  1 (0.7)  24 (16.7)  1 (0.7)  3 (2.1)  0 (0)  1 (0.7) |
| --- | --- |

TAVI

| 23 mm  24 mm  25 mm  26 mm  27 mm  29 mm  34 mm | 7 (17.5)  2 (5.0)  2 (5.0)  13 (32.5)  3 (7.5)  12 (30.0)  1 (2.5) |
| --- | --- |

SAVR: surgical aortic valve replacement; TAVI: transcatheter aortic valve implantation.

**Online Resource 4. SAVR approach.**

| Sternotomy | 132 (96.4) |
| --- | --- |
| Mini-sternotomy | 5 (3.4) |

SAVR: surgical aortic valve replacement

**Online Resource 5. TAVI access.**

| Transfemoral | 40 (97.6) |
| --- | --- |
| Transaxilar | 1 (2.4) |

TAVI: transcatheter aortic valve implantation.

**Online Resource 6. Last postprocedural echocardiographic follow-up before IE diagnosis.**

|  | **SAVR**  **(n=169)** | **TAVI**  **(n=41)** | **p** |
| --- | --- | --- | --- |
| Time from valve implantation (days) | 724 (78.5-1825) | 125 (36-356) | **0.001** |
| Leaflet thickening or calcification | 18 (18.8) | 2 (7.7) | 0.177 |
| AVA (cm^2^) | 2.0 (1.2-2.1) | 1.5 (1.3-1.6) | 0.506 |
| V max (m/s) | 2.4 (2.1-2.8) | 2.3 (1.8-2.8) | 0.241 |
| Mean pressure gradient (mmHg) | 14.9 (10.6-22) | 12.8 (8.9-16.5) | 0.091 |
| Intra-prosthetic regurgitation   - Mild - Moderate - Severe | 16 (15.8)  4 (4.0)  0 (0) | 7 (26.9)  0 (0)  0 (0) | 0.277 |
| Peri-prosthetic regurgitation   - Mild - Moderate - Severe | 7 (6.9)  0 (0)  4 (4.0) | 6 (23.1)  3 (11.5)  0 (0) | **0.001** |
| Time from last follow-up to admission for IE (days) | 103.5 (18-383) | 95 (39-178) | 0.924 |

Values are presented as frequency and percentage or median and interquartile ranges. Bold values are significant.

AVA: aortic valve area; IE: infective endocarditis; SAVR: surgical aortic valve replacement; TAVI: transcatheter aortic valve implantation; V max: peak velocity.

**Online Resource 7. Causative microorganisms.**

|  | **IE-SAVR**  **(n=169)** | **IE-TAVI**  **(n=41)** | **p** |
| --- | --- | --- | --- |
| *Staphylococcus epidermidis* | 45 (26.6) | 11 (26.8) | 0.979 |
| Enterococcus spp. | 31 (18.3) | 10 (24.4) | 0.381 |
| *Staphylococcus aureus* | 19 (11.2) | 9 (22.0) | 0.070 |
| *Viridans* group streptococci | 26 (15.4) | 3 (7.3) | 0.179 |
| *Streptococcus gallolyticus* | 11 (6.5) | 3 (7.3) | 0.852 |
| Polymicrobial | 8 (4.7) | 1 (2.4) | 0.515 |
| Other streptococci | 3 (1.8) | 0 (0) | 0.390 |
| Fungi | 7 (4.1) | 0 (0) | 0.185 |
| Gram-negative bacilli | 2 (1.2) | 0 (0) | 0.484 |
| Anaerobia | 1 (0.6) | 1 (2.4) | 0.275 |
| HACEK group | 2 (1.2) | 0 (0) | 0.484 |
| Other | 2 (1.2) | 1 (2.4) | 0.543 |
| Culture-negative endocarditis | 12 (7.1) | 2 (4.9) | 0.609 |

Values are presented as frequency and percentage or median and interquartile ranges. Bold values are significant.

HACEK group includes *Haemophilus* species, *Actinobacillus actinomycetemcomitans*, *Cardiobacterium hominis*, *Eikenella corrodens,* and *Kingella kingae.*

IE-SAVR: infective endocarditis after surgical aortic valve replacement with a biological prosthetic valve; infective endocarditis-TAVI: IE after transcatheter aortic valve implantation.

**Online Resource 8. Echocardiographic findings according to TAVI design.**

|  | **Balloon-expandable**  **(n=15)** | **Self-expandable**  **(n=22)** | **Other designs**  **(n=3)** | **p** |
| --- | --- | --- | --- | --- |
| Vegetation | 11 (73.3) | 12 (54.6) | 3 (100.0) | 0.183 |
| Vegetation diameter (mm) | 15 (10-24) | 12 (8-16) | 8 (6-12) | 0.201 |
| Periannular complications   - Abscess - Pseudoaneurysm | 2 (13.3)  1 (6.7)  1 (6.7) | 5 (22.7)  5 (22.7)  1 (4.6) | 1 (33.3)  1 (33.3)  0 (0) | 0.773  0.492  0.957 |
| Abscess diameter (mm) | 5 (5-5) | 6 (5-8) | 8 (8-8) | 0.553 |
| Pseudoaneurysm diameter (mm) | 10 (10-10) | 3.5 (3.5-3.5) | - | 0.317 |

Values are presented as frequency and percentage or median and interquartile ranges.

**Online Resource 9. Findings on other imaging tests**

|  | **IE-SAVR**  **(n=169)** | **IE-TAVI**  **(n=41)** | **p** |
| --- | --- | --- | --- |
| IE-related findings on other imaging tests | | | |
| Splenomegaly | 4^a^ | 0 | - |
| Hepatomegaly | 2^b^ | 0 | - |
| Splenic infarction | 27^c^ | 4^d^ | - |
| Splenic abscess | 3^e^ | 0 | - |
| Hepatic infarction | 1^f^ | 0 | - |
| Renal infarction | 2^g^ | 1^h^ | - |
| Lower limbs embolism | 2^i^ | 0 | - |
| Spondylodiscitis | 5^j^ | 2^k^ | - |
| Sacroiliac arthritis | 1^l^ | 0 | - |
| Arthritis in another location | 0 | 1^m^ | - |
| Pulmonary embolism | 0 | 1^n^ | - |
| Cerebral embolism | 26^o^ | 7^p^ | - |
| Intracranial bleeding | 5^q^ | 0 | - |
| Subarachnoid bleeding | 3^r^ | 1^s^ | - |
| Infectious aneurysms | 3^t^ | 0 | - |
| Other embolisms | 2^u^ | 0 | - |
| Diagnostic yield of other imaging techniques | | | |
| Abdominal ultrasound   - Performed - IE-related findings | 56 (33.1)  15 (8.9) | 6 (14.6)  2 (4.9) | 0.118 |
| Thoracic/Abdominal CT   - Performed - IE-related findings | 55 (32.5)  27 (16.0) | 13 (31.7)  4 (9.6) | 0.496 |
| Cerebral CT   - Performed - IE-related findings | 77 (45.6)  25 (14.8) | 16 (39.0)  7 (17.1) | 0.586 |
| Cerebral magnetic resonance   - Performed - IE-related findings | 13 (7.7)  7 (4.1) | 2 (4.9)  2 (4.9) | 0.371 |
| Cerebral arteriography   - Performed - IE-related findings | 11 (6.5)  1 (0.6) | 1 (2.4)  0 (0) | 0.588 |
| PET/CT   - Performed - IE-related findings - Radionuclide uptake at the aortic prosthetic valve | 69 (40.8)  42 (24.9)  32 (46.3) | 19 (46.3)  14 (34.2)  12 (63.2) | 0.674 |

Values are presented as frequency and percentage.

^a^ 3 cases detected on abdominal ultrasound. 1 case detected on abdominal ultrasound and CT.

^b^ 1 case detected on abdominal ultrasound. 1 case detected on abdominal ultrasound and CT.

^c^ 4 cases detected on abdominal ultrasound and CT. 1 case detected on abdominal ultrasound. 1 case detected on abdominal ultrasound, CT and PET/CT. 12 cases detected on CT. 2 cases detected on CT and PET/CT. 7 cases detected on PET/CT.

^d^ 1 case detected on abdominal ultrasound. 2 cases detected on CT. 1 case detected on abdominal ultrasound and CT.

^e^ 2 cases detected on CT. 1 case detected on CT and abdominal ultrasound.

^f^ 1 case detected on abdominal ultrasound.

^g^ 2 cases detected on CT.

^h^ 1 case detected on PET/CT.

^i^ 1 case detected on CT. 1 case detected on PET/CT.

^j^ 4 cases detected on PET/CT. 1 case detected on CT and PET/CT.

^k^ 2 cases detected on PET/CT.

^l^ 1 case detected on CT and PET/CT.

^m^ 1 case detected on PET/CT.

^n^ 1 case detected on CT and PET/CT.

^o^ 19 cases detected on CT. 5 cases detected on CMR. 2 cases detected on PET/CT.

^p^ 6 cases detected on CT. 1 case detected on CMR. 1 case detected on CT and CMR.

^q^ 3 cases detected on CT. 2 cases detected on CMR.

^r^ 3 cases detected on CT.

^s^ 1 case detected on CT.

^t^ 2 cases detected on CMR. 1 case detected on CT.

^u^ 2 cases detected on PET/CT in a non-specified location.

CMR: cerebral magnetic resonance; CT: computed tomography; IE: infective endocarditis; IE-SAVR: IE after surgical aortic valve replacement with a biological prosthetic valve; IE-TAVI: IE after transcatheter aortic valve implantation; PET: positron emission tomography.

**Online Resource 10. Other cardiac structures compromised by IE (apart from SAVR or TAVI).**

|  | SAVR | TAVI | p |
| --- | --- | --- | --- |
| Native mitral valve | 14 (8.3) | 5 (12.2) | 0.866 |
| Native tricuspid valve | 3 (1.7) | 0 (0) |  |
| Mechanical prosthetic mitral valve | 1 (0.6) | 0 (0) |  |
| Biological prosthetic mitral valve | 5 (3.0) | 0 (0) |  |
| CIED | 4 (2.4) | 0 (0) |  |
| Intravascular catheter | 1 (0.6) | 0 (0) |  |
| Supracoronary tube graft | 1 (0.6) | 0 (0) |  |
| Aortitis | 3 (1.7) | 0 (0) |  |
| Other | 2 (1.2) | 0 (0) |  |

CIED: cardiac implantable electronic device; IE: infective endocarditis; IE-SAVR: IE after surgical aortic valve replacement with a biological prosthetic valve; IE-TAVI: IE after transcatheter aortic valve implantation.

**Online Resource 11. Analysis of the clinical and microbiological profile of EI-TAVI patients with indication for cardiac surgery who were not intervened (n=19)**

|  | **Discharged alive**  **(n=7)** | **Death during hospitalization**  **(n=12)** | **p** |
| --- | --- | --- | --- |
| Age at IE diagnosis | 82.4 (1.6) | 80.0 (1.9) | 0.418 |
| Heart failure during admission | 3 (42.9) | 11 (91.7) | **0.020** |
| Periannular complications | 1 (14.3) | 5 (41.7) | 0.216 |
| Septic shock during admission | 0 (0) | 6 (50.0) | **0.024** |
| Stroke during admission | 1 (14.3) | 4 (33.3) | 0.363 |
| Acute renal failure | 3 (42.9) | 9 (75.0) | 0.161 |
| Causative microorganism:   - *S. aureus* - *S. epidermidis* - *E. faecalis* - *S. gallolyticus* - Negative cultures | 2 (28.5)  0 (0)  3 (42.3)  1 (14.3)  1 (14.3) | 3 (25.0)  5 (41.7)  1 (8.3)  1 (8.3)  1 (8.3) | 0.314 |

Values are presented as frequency and percentage or median and interquartile ranges. Bold values are significant.

IE: infective endocarditis.
